# ADVERSE CHILDHOOD EXPERIENCES AND SOCIAL PARTICIPATION ON FRAILTY STATE TRANSITIONS: A 10-YEAR PROSPECTIVE COHORT

**DOI:** 10.1093/geroni/igae098.4320

**Published:** 2024-12-31

**Authors:** Jiajia Li, Heming Pei, Ning Kang, Gong Chen, Lijun Pei

**Affiliations:** Peking University, Beijing, Beijing, China (People’s Republic); Columbia University, New York, New York, United States; Peking University, Beijing, Beijing, China (People’s Republic); Peking University, Beijing, Beijing, China (People’s Republic); Peking University, Beijing, Beijing, China (People’s Republic)

## Abstract

**Background:**

Adverse childhood experiences (ACEs) is associated with frailty, while the association with frailty state transitions and the role of social participation remain unclear. This study aimed to investigate the association between ACEs and frailty state transition, alongside the moderation effect of social participation

**methods:**

Data from 9,621 adults aged 45+ from the CHARLS (2011-2020) were analyzed. Frailty was measured with the frailty index, while ACEs and social participation were measured with a validated questionnaire. The association between ACEs and frailty state transitions was estimated with multi-state models. Interaction analysis was used to examine the moderation effects of social participation.

**Results:**

Participants exposed to higher ACEs levels (≥ 4) were associated with an increased risk of forward transition (robust to pre-frail, HR=1.36, 95%CI: 1.21-1.53; prefrail to frail, HR=1.38, 95%CI: 1.17 - 1.62) and decreased risk of backward transition (pre-frail to robust, HR = 0.64, 95%CI: 0.54 – 0.76). Additionally, participants with social participation were associated with increased risk of backward transition (frail to pre-frail, HR = 1.14, 95%CI: 1.02 – 1.27). Social participation moderated the association between ACEs exposure and frailty (P for interaction < 0.05), while participants with lower ACEs exposure levels and social participation were associated with an increased risk of transition from frail to pre-frail (ACEs levels =1, HR = 1.57, 95%CI: 1.06 – 2.32; ACEs levels =2, HR = 1.49, 95%CI: 1.02 – 2.17).

**Conclusion:**

High levels of ACEs exposure were associated with a greater likelihood of adverse frailty development. Older adults with ACEs exposure might benefit from intervention strategies to improve social participation.

